# Growth patterns in patients with mucopolysaccharidosis VII

**DOI:** 10.1016/j.ymgmr.2023.100987

**Published:** 2023-06-26

**Authors:** Adriana M. Montaño, Agnieszka Różdżyńska-Świątkowska, Agnieszka Jurecka, Antonio Nino Ramirez, Lin Zhang, Deborah Marsden, Raymond Y. Wang, Paul Harmatz

**Affiliations:** a Department of Pediatrics, and Biochemistry and Molecular Biology, School of Medicine, Saint Louis University, St. Louis, MO, USA; b Anthropology Laboratory, Children's Memorial Health Institute, Warsaw, Poland; c Ultragenyx Pharmaceutical Inc., Novato, CA, USA; d Division of Metabolic Disorders, Children's Hospital of Orange County, USA; e Division of Pediatrics, University of California-Irvine School of Medicine, Orange, CA, USA; f UCSF Benioff Children's Hospital, Oakland, CA, USA

**Keywords:** MPS VII, Height, Weight, BMI, Growth

## Abstract

**Objective:**

This study assessed growth patterns in patients with mucopolysaccharidosis (MPS) VII before enzyme replacement therapy.

**Methods:**

Height, weight, and body mass index (BMI) measurements and *Z*-scores from patients from three clinical studies were compared with those from CDC healthy population growth charts. Relationships with age/sex and history of non-immune hydrops fetalis (NIHF) were assessed by linear regression and ANOVA, respectively.

**Results:**

Among 20 enrolled patients with MPS VII, height *Z*-scores were near normal until 1 year of age but declined thereafter, particularly among males. There was no consistent pattern in weight *Z*-score. BMI *Z*-scores were above normal and increased slightly with age among males and were slightly below normal among females. Male patients with a history of NIHF had greater declines in height and weight *Z*-scores over time versus males without history of NIHF. There was no clear effect of NIHF history on height and weight *Z*-scores in female patients.

**Conclusions:**

In patients with MPS VII, declines in height *Z*-score began early in life, particularly among males, while changes in BMI varied by sex. Patients with MPS VII and a history of NIHF had greater declines in height *Z*-score with age than did patients without a history of NIHF.

**Clinical trial registration:** This retrospective analysis included patients enrolled in an open-label phase 2 study (UX003-CL203; ClinicalTrials.gov, NCT02418455), a randomized, placebo-controlled, blind-start phase 3 study (UX003-CL301; ClinicalTrials.gov, NCT02230566), or its open-label, long-term extension (UX003-CL202; ClinicalTrials.gov, NCT02432144). Requests for individual de-identified participant data and the clinical study report from this study are available to researchers providing a methodologically sound proposal that is in accordance with the Ultragenyx data sharing commitment. To gain access, data requestors will need to sign a data access and use agreement. Data will be shared via secured portal. The study protocol and statistical analysis plan for this study are available on the relevant clinical trial registry websites with the tabulated results.

## 1 Introduction

Mucopolysaccharidosis VII (MPS VII; Sly syndrome) is an ultra-rare lysosomal storage disorder characterized by deficiency in β-glucuronidase, resulting in the accumulation of the glycosaminoglycans (GAGs) chondroitin 4-sulfate (C4S), chondroitin 6-sulfate, dermatan sulfate, and heparan sulfate [1]. MPS VII presents a continuous clinical spectrum that can be phenotypically classified into three groups: neonatal with a history of prenatal non-immune hydrops fetalis (NIHF) that may have persistent features, neonatal without a history of NIHF, and adolescent [2,3]. NIHF is a severe, prenatal manifestation of MPS VII that often results in fetal demise or early infant death [2,3]. Skeletal abnormalities are universal in mucopolysaccharidoses [4], and patients with infantile and adolescent forms of MPS VII commonly present with short stature, growth impairment, and skeletal dysplasia [2]. In most types of MPS, normal or accelerated growth is typically observed from birth until the first 2–4 years of age, when growth velocity decreases to below normal [2,5].

The mechanisms of poor growth in MPS are unclear but may be related to chondrocyte and osteoblast GAG accumulation, resulting in decreased matrix deposition, disorganized growth plate structure, and impaired osteoblast osteoid synthesis [6,7]. GAG storage induces a complex sequence of molecular abnormalities, such as inflammation, chondrocyte apoptosis, and synovial membrane hyperplasia, that result in poorly organized and metabolically abnormal connective tissue matrices [[8], [9], [10], [11], [12]]. A preclinical study in mice with MPS VII demonstrated that shortened bones may result from the accumulation of GAGs (eg, C4S) in the growth plate, resulting in reduced expression of leukemia inhibitory factor and decreased STAT3 tyrosine phosphorylation, and consequently, reduced chondrocyte proliferation [13]. There is preclinical and clinical evidence of prenatal accumulation of GAGs in chondrocytes in MPS [[14], [15], [16], [17]]. Delayed development of secondary ossification centers in the vertebrae and long bones is one of the earliest skeletal abnormalities to manifest in a naturally occurring canine model of MPS VII [17,18]. The resulting cartilaginous lesions (epiphyseal cartilage that fails to transition to bone) persist beyond skeletal maturity and likely contribute to progressive skeletal dysplasia [[19], [20], [21]].

Monitoring growth patterns, including height, is crucial for the assessment of disease progression and therapeutic efficacy in MPS [5]; however, investigations of growth patterns in patients with MPS VII have been rare. To our knowledge, the only reported longitudinal data on growth patterns in MPS VII are from a relatively small survey (*n* = 12) that described changes in height, weight, and body mass index (BMI) from birth to age 20 years compared with age-matched controls [2]. This larger retrospective analysis of clinical studies evaluated growth patterns among patients with MPS VII from birth until introduction of enzyme replacement therapy (ERT) compared with a healthy population and included assessments of the relationship of sex and age with standing height or recumbent length and with BMI. The impact of history of NIHF on growth patterns was also determined.

## 2 Methods

### 2.1 Patients

This retrospective analysis included patients with MPS VII who were enrolled in an open-label phase 2 study (UX003-CL203; ClinicalTrials.gov, NCT02418455), a randomized, placebo-controlled, blind-start phase 3 study (UX003-CL301; ClinicalTrials.gov, NCT02230566), or its open-label, long-term extension (UX003-CL202; ClinicalTrials.gov, NCT02432144). CL203 was conducted at five centers in the United States, Spain, and Portugal. CL301 was conducted at four centers in the United States. CL202 was conducted at seven centers in the United States, Brazil, Mexico, and Portugal.

As previously described [[22], [23], [24]], eligible patients were aged <5 years (CL203) or 5–35 years (CL202 and CL301), had confirmed diagnosis of MPS VII based on leukocyte or fibroblast b-glucuronidase (GUS) enzyme assay or *GUSB* genetic testing; had urinary glycosaminoglycan (uGAG) excretion ≥3-fold over the mean normal for age (CL202 and CL301); had apparent signs of lysosomal storage disease per the investigator (enlarged liver and spleen, limited joint mobility, airway obstruction or pulmonary problems, or limited mobility while ambulatory) (CL202 and CL301); had no prior bone marrow or stem cell transplant; and had no major surgery within three months before study enrollment (CL301).

Institutional review boards/ethics committees at each site approved the study protocols. Parents/guardians provided written informed consent for children to participate, and when appropriate, the patients' assents were obtained before proceeding. Individual patient data were de-identified before use in this manuscript. All studies adhered to the principles of the Declaration of Helsinki.

### 2.2 Assessments

Using growth charts in medical histories, this retrospective analysis of studies CL203, CL301, and CL202 assessed patient height, weight, and BMI data before initiation of ERT with vestronidase alfa. Anthropometric measurements were taken repeatedly using standard techniques and included body height and weight. For all three studies, Clinical Evaluator Manuals with specific training requirements for measuring height were provided. All three studies measured standing height. In addition, studies CL202 and CL301 measured seated height, and study CL203 (patients aged <5 years) measured recumbent length. Each site used their own equipment that were regularly calibrated. BMI was assessed as previously described [5]. PolyPhen-2 [38] was used to predict the possible impact of missense mutations on the structure and function of the human GUSB protein.

### 2.3 Statistical analysis

Demographics and baseline clinical characteristics were summarized descriptively. Height, weight, and BMI were evaluated versus the height, weight, and BMI percentiles of a healthy population using the Centers for Disease Control (CDC) and National Center for Health Statistics Clinical Growth Chart [25]. Linear regression was used to investigate the trend of growth (height, weight, and BMI *Z*-scores) with age among male and female patients with MPS VII compared with a healthy population. A *t*-test was used to assess the difference in height *Z*-scores of patients with MPS VII versus the height *Z*-scores of the healthy population in subgroups by sex and age. Analysis of variance (ANOVA) with patient as a random effect was used to determine significant differences in variables between patients with or without a history of NIHF.

## 3 Results

### 3.1 Patients

Overall, 20 patients from the three clinical studies (CL203, *n* = 8; CL301/CL202, *n* = 12) were included in the analysis of growth patterns before treatment with vestronidase alfa. The mean age of the cohort at diagnosis of MPS VII was 4.01 years (SD, 1.08 years), ranging from birth up to 16.0 years (Supplemental Table 1).

The majority of patients were female and white. At enrollment, 12 (60%) patients had at least one *GUSB* mutation, the most frequent of which was c.526C > T (*n* = 3; Supplemental Table 2).

Five (25%) patients had a history of NIHF, and all patients had skeletal manifestations (Supplemental Table 2).

### 3.2 Relationship between age, sex, and growth in MPS VII

**Fig. 1:**
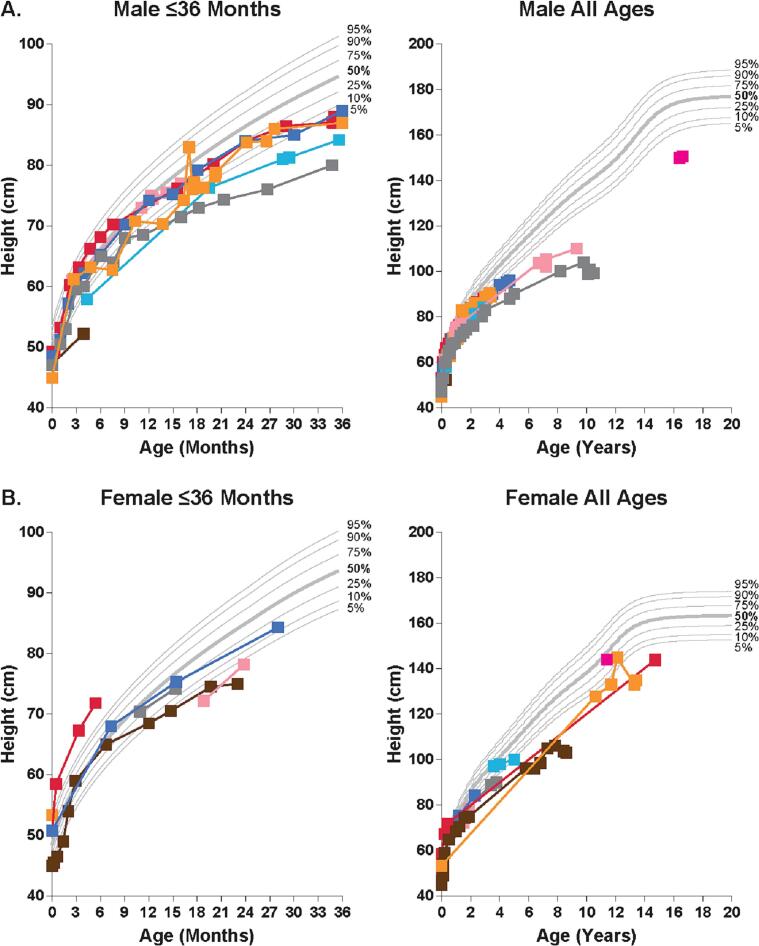
Change over time in height in patients with MPS VII compared with a healthy population. Height was assessed in male (A) and female (B) patients with MPS VII aged ≤36 months and all ages versus the height percentiles of the healthy population using the Centers for Disease Control and National Center for Health Statistics Clinical Growth Chart. Each data represents a single measurement; colors show measurements over time in a single patient.

Compared with the healthy CDC population, height in both male and female patients with MPS VII appeared normal during the first year of life but then declined to below normal after 1 year of age, particularly among male patients (Fig. 1).

**Fig. 2:**
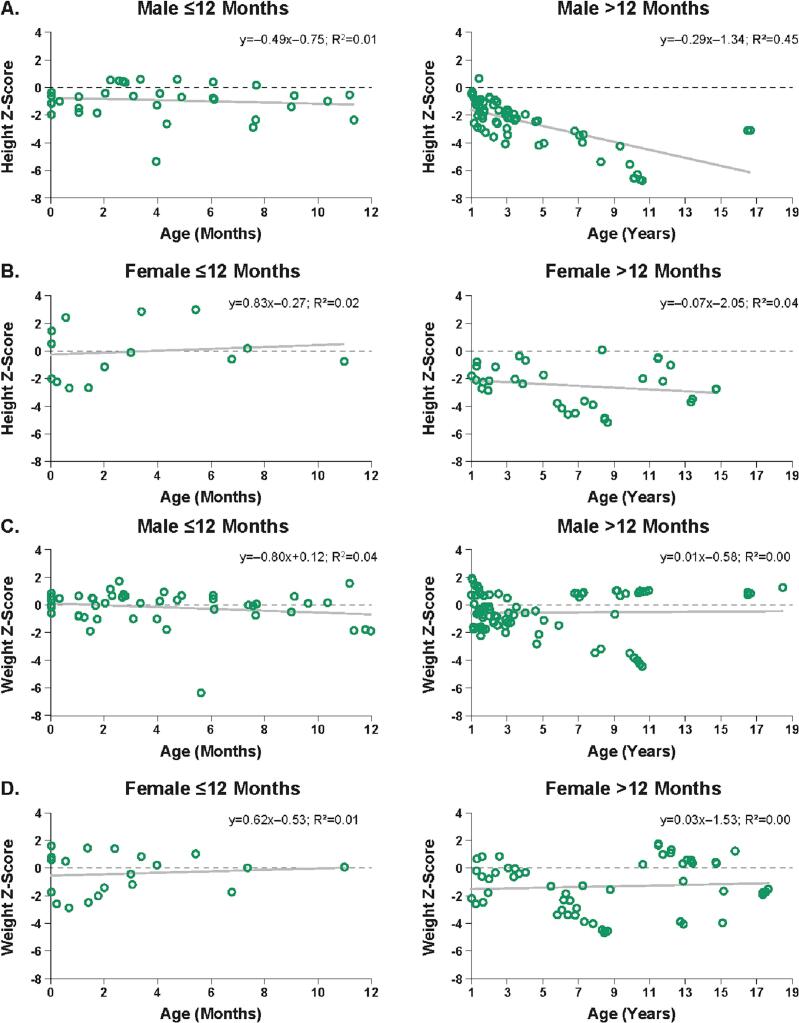
Relationship of height and weight with age in patients with MPS VII. Linear regression analysis of height *Z*-score in male (A) and female (B) patients with MPS VII aged ≤12 months and > 12 months. Linear regression of weight Z-score in male (C) and female (D) patients with MPS VII aged ≤12 months and > 12 months.

During the first year of life, male patients with MPS VII had a mean height *Z*-score slightly below that of the healthy population (y = −0.49× − 0.75; R^2^ = 0.01), whereas female patients had a mean height *Z*-score approximately equal to that of the healthy population (y = 0.83× − 0.27; R^2^ = 0.02; Fig. 2A,B).

After the first year of life, the mean height *Z*-score of male patients with MPS VII markedly decreased with age to much further below normal (y = −0.29× − 1.34; R^2^ = 0.45) but the mean height *Z*-score of female patients only slightly decreased with age (y = −0.07× − 2.05; R^2^ = 0.04). In a subgroup analysis, the greatest differences in height *Z*-score among patients with MPS VII, compared with the healthy population, appeared to be among males aged 1–3 years (*P* = 0.003) and 4–6 years (*P* = 0.040) and females aged 1–3 years (*P* = 0.031) and ≥ 10 years (*P* = 0.030; Supplemental Table 3); however, several subgroups were limited by small sample sizes.

**Fig. 3:**
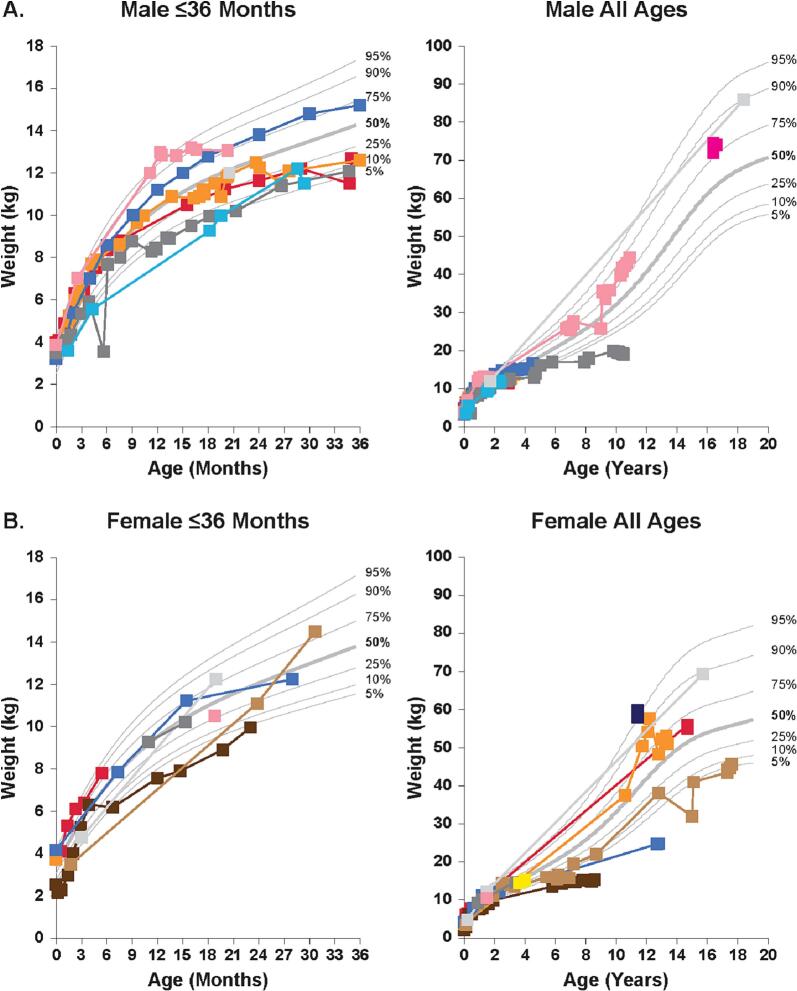
Change over time in weight in patients with MPS VII compared with a healthy population. Weight was assessed in male (A) and female (B) patients with MPS VII aged ≤36 months and all ages versus the height percentiles of the healthy population using the Centers for Disease Control and National Center for Health Statistics Clinical Growth Chart. Each data represents a single measurement; colors show measurements over time in a single patient.

Compared with the healthy population, no consistent patterns in weight were exhibited for either sex in patients with MPS VII. The weight of patients with MPS VII varied widely, ranging from below the 5th percentile weight of the healthy population up to the 95th percentile weight of the healthy population (Fig. 3).

During the first year of life, the mean weight *Z*-score, compared with that of the healthy population, was approximately normal in male patients with MPS VII (y = −0.80× + 0.12; R^2^ = 0.04) and in female patients (y = 0.62× − 0.53; R^2^ = 0.01; Fig. 2C,D). After the first year of life, the mean weight *Z*-score remained steady and was slightly below normal in male patients (y = 0.01× − 0.58; R^2^ = 0.00) and in female patients (y = 0.03× − 1.53; R^2^ = 0.00).

Compared with the healthy population, the BMI of patients with MPS VII was slightly above normal in earlier years in males and varied over time among females (Supplemental Fig. 1A,B). The mean BMI *Z*-score was above that of the healthy population in male patients (y = 0.07× + 0.85; R^2^ = 0.10) and was slightly below normal in female patients (y = 0.01× − 0.64; R^2^ = 0.00; Supplemental Fig. 2).

### 3.3 Relationship between growth and non-immune hydrops fetalis in MPS VII

**Fig. 4:**
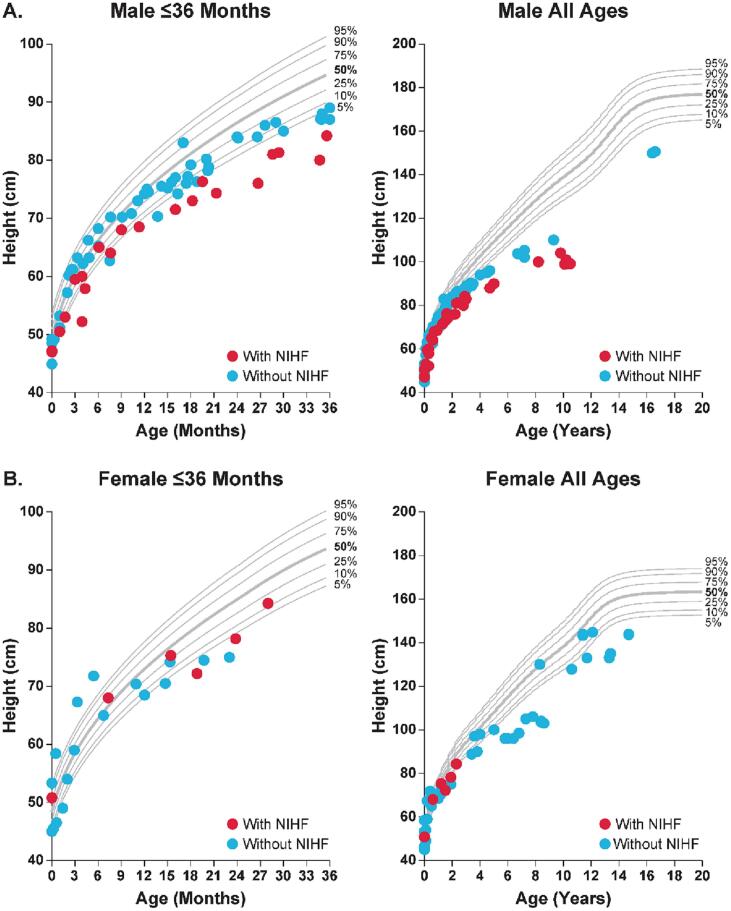
Effect of history of NIHF on the change over time in height in patients with MPS VII. Height was assessed in male (A) and female (B) patients with MPS VII aged ≤36 months and all ages versus the height percentiles of the healthy population using the Centers for Disease Control and National Center for Health Statistics Clinical Growth Chart. Each red dot represents a single height measurement in a patient with a history of NIHF; each blue dot represents a single height measurement in a patient without a history of NIHF. MPS, mucopolysaccharidosis; NIHF, non-immune hydrops fetalis.

Compared with the healthy population, male patients with MPS VII and a history of NIHF appeared to have greater declines in height *Z*-score than did male patients without a history of NIHF (Fig. 4).

There was no apparent pattern in height by history of NIHF among female patients due to the small sample size. Using an ANOVA model for the total population with patient as a random effect, patients aged ≥10 years were the only age group with a significant reduction in height *Z*-score when comparing history of NIHF versus no history of NIHF (mean *Z*-score, −4.23 versus −1.61; *P* = 0.010; Supplemental Table 4). However, some age subgroups assessed with the ANOVA model were limited by small sample sizes.

**Fig. 5:**
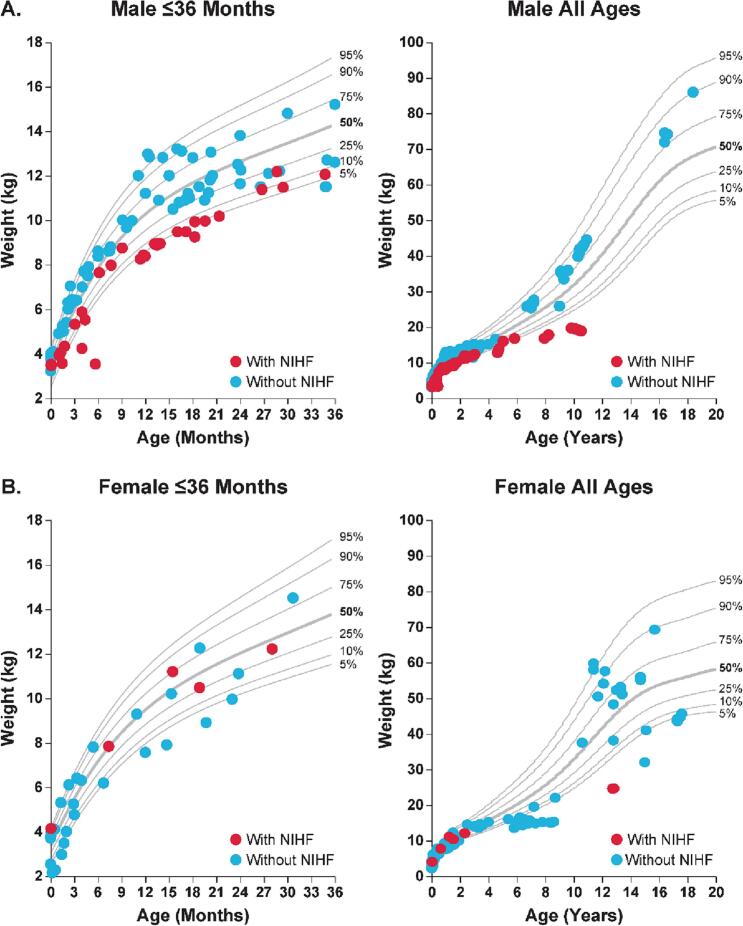
Effect of history of NIHF on the change over time in weight in patients with MPS VII. Weight was assessed in male (A) and female (B) patients with MPS VII aged ≤36 months and all ages versus the height percentiles of the healthy population using the Centers for Disease Control and National Center for Health Statistics Clinical Growth Chart. Each red dot represents a single weight measurement in a patient with a history of NIHF; each blue dot represents a single weight measurement in a patient without a history of NIHF. MPS, mucopolysaccharidosis; NIHF, non-immune hydrops fetalis.

Compared with the healthy population, weight was lower among male patients with MPS VII and a history of NIHF compared with males without a history of NIHF (Fig. 5).

There was no clear pattern in weight among female patients with MPS VII based on history of NIHF. Using the ANOVA model for the total population, significant differences in weight *Z*-scores when comparing history of NIHF to no history of NIHF were seen only for patients aged >3 to 6 months (mean *Z*-score, −4.07 versus 0.18; *P* < 0.0001) and patients aged ≥10 years (mean *Z*-score, −3.85 versus 0.70; *P* < 0.0001; Supplemental Table 4). However, some age groups were limited by small sample sizes.

Compared with the healthy population, there was no apparent difference in BMI patients with MPS VII when comparing those with a history of NIHF versus those without a history of NIHF (Supplemental Fig. 1C,D). Using the ANOVA model for the total population, BMI *Z*-scores were not significantly different for any of the age groups when comparing history of NIHF and no history of NIHF (Supplemental Table 4).

## 4 Discussion

Published data on growth patterns in patients with MPS VII are limited due to the rarity of the disease. To our knowledge, this is the largest (*n* = 20) longitudinal analysis of growth patterns among patients with MPS VII before initiation of treatment with ERT. In this population of patients with MPS VII, the majority of whom were white, height *Z*-scores were near normal during the first year of life but were below normal thereafter, particularly in male patients, among whom there was a substantial decrease in height *Z*-scores versus the healthy CDC reference population. These results are similar to those of our previous smaller survey, in which 12 patients with MPS VII had normal length *Z*-scores until 18–24 months of age, when height Z-scores decreased to below normal in males (mean 18-year *Z*-score, −3.3 vs CDC control) and in females (mean 18-year *Z*-score, −7.8) [2]. However, in that study, the most rapid declines in height *Z*-score (ie, below the 5th percentile of the healthy population) occurred after the age of 13 years in males and after the age of 2 years in females [2]. In a study of the growth charts of 354 patients with MPS IVA, height *Z*-scores were normal among male and females until 2 years of age but decreased to below normal beginning at 4 years of age, with decreased growth velocity (ie, height gained per year) after the first year of life [26]. A similar pattern of initially normal or increased height, height *Z*-scores, and/or growth velocity versus those of healthy controls, followed by declines to below normal height, has been observed in patients with MPS I, II, IVA, and VI (Supplemental Table 5) [[27], [28], [29], [30], [31], [32]].

In this study, weight varied widely among patients with MPS VII, ranging from below the 5th percentile of the healthy population weight up to the 95th percentile of the healthy population weight. For both male and female patients, weight *Z*-scores were approximately normal during the first year of life and then slightly below normal thereafter. Across all ages, BMI *Z*-scores in patients with MPS VII were slightly above normal among males and near among females. In our earlier survey, BMI was largely normal but variable among 13 adult male patients with MPS VII and below normal to normal BMI in seven of eight adult female patients with MPS VII [2]. The difference in BMI results of this study versus the previous survey could be attributed, at least in part, to the small numbers of patients in certain age/sex groups, as well as differences in the ages of patients (ie, <5 years versus 5–35 years). However, consistent with the results of the present study, increased BMI *Z*-scores versus healthy controls (ie, overweight or obese) have been reported among adult patients with treated and untreated MPS IVA, particularly among males [26,33]. Above average childhood BMI *Z*-score has also been reported among patients with MPS II [27,28,34].

In a recent case review study, Holtz et al. reported that 72 of 148 (48.6%) patients with MPS VII presented with NIHF, either *in utero* or at delivery, and that 63.9% of NIHF cases resulted in intrauterine fetal demise or did not survive longer than 6 months of age [3]. We previously reported that presence of neonatal NIHF does not by itself predict the eventual severity of the disease among patients who survive [2]. In the current study, we demonstrate a decrease in height and weight *Z*-scores among male patients with MPS VII and a history of NIHF but not among female patients with MPS VII and a history of NIHF. Using the ANOVA model, the greatest differences in height and weight *Z*-scores when comparing history of NIHF versus no history of NIHF occurred among the oldest patients (ie, aged ≥10 years). In contrast, there was no clear pattern in BMI *Z*-score in patients with a history of NIHF compared with patients without a history of NIHF. However, because of small sample sizes in several age subgroups, these ANOVA results should be interpretated with caution.

There is ample evidence that the accumulation of GAGs (eg, dermatan sulfate and chondroitin sulfate) contributes to growth plate abnormalities in MPS, including MPS VII [6,7,[13], [14], [15], [16], [17],^35^]. Although the relationship between GAG levels and NIHF is unclear, elevated GAG levels have been detected as early as 21 weeks of pregnancy in the amniotic fluid of a fetus with MPS VII and NIHF [36]. There is some preclinical and clinical evidence indicating that reduction of uGAG levels could be useful in estimating efficacy of ERT in patients with MPS, including MPS VII [37]. Unfortunately, uGAG data were insufficient for longitudinal comparison with growth patterns in this study.

## 5 Conclusion

In this population of patients with MPS VII not yet treated with ERT, noteworthy patterns in growth were observed. Among male patients with MPS VII, compared with a healthy population, height *Z*-scores were near normal for the first year of life but declined to significantly below normal thereafter, whereas BMI *Z*-scores were slightly above normal throughout the observation period. Notably, male patients with MPS VII who had a history of NIHF had markedly decreased height and weight *Z*-scores compared with patients without a history of NIHF. The underlying causes of these growth patterns among patients with MPS VII remain unknown. Despite being limited in some cases by small sample sizes, these data provide direct clinical evidence of the physiologic impact of changes at the growth plate due to GAG accumulation in MPS VII. Growth evaluations could therefore be helpful for monitoring the clinical efficacy of available and future therapies for MPS VII.

## Funding/support

This work was funded by 10.13039/100013220Ultragenyx Pharmaceutical Inc.

## Role of funder/sponsor

The sponsor contributed to the design and analysis of the included clinical studies.

## Article summary

This study provides longitudinal evidence of abnormal growth patterns in patients with MPS VII due to changes at the growth plate resulting from glycosaminoglycan accumulation.

## What's known on this subject

Only one relatively small (*n* = 12) longitudinal assessment of growth patterns in MPS VII has been published as part of the natural history of the disease. Additionally, a comprehensive study showed that almost half of MPS VII patients present hydrops fetalis.

## What this study adds

Height *Z*-scores in treatment-naïve patients (*n* = 20) with MPS VII were near normal until age 1 but declined thereafter (especially in males), whereas body mass index *Z*-scores were above average. Male patients with NIHF history had greater height/weight *Z*-score declines.

## Contributors statement page

Drs Adriana M. Montaño and Agnieszka Jurecka contributed to the conceptualization and design of the included studies.

Drs Agnieszka Różdżyńska-Świątkowska, Raymond Y. Wang, and Paul Harmatz contributed to the clinical investigations of the included clinical studies.

Drs Adriana M. Montaño, Agnieszka Jurecka, Raymond Y. Wang, and Paul Harmatz contributed to the administration of the included clinical studies.

Drs Adriana M. Montaño, Agnieszka Różdżyńska-Świątkowska, Raymond Y. Wang, and Paul Harmatz contributed to the supervision of the included clinical studies.

Drs Adriana M. Montaño, Agnieszka Różdżyńska-Świątkowska, Agnieszka Jurecka, Antonio Nino Ramirez, Lin Zhang, Deborah Marsden, Raymond Y. Wang, and Paul Harmatz analyzed and interpreted the data.

All authors drafted or revised the manuscript critically.

## Declaration of Competing Interest

Adriana M. Montaño has received research support from 10.13039/100013220Ultragenyx Pharmaceutical Inc. Agnieszka Różdżyńska-Świątkowska declares no conflicts of interest. Agnieszka Jurecka, Antonio Nino Ramirez, Lin Zhang, and Deborah Marsden are employees of and own stock in Ultragenyx Pharmaceutical Inc. Raymond Y. Wang has received research support from Ultragenyx Pharmaceutical Inc. Paul Harmatz has received research and consulting support from Ultragenyx Pharmaceutical Inc.

## Data Availability

Data will be made available on request.

## Appendix A Supplementary data

Supplementary material Image 1
