# Supplementary material for: Growth patterns in patients with mucopolysaccharidosis VII
Source: Mol Genet Metab Rep. 2023 Jun 26;36:100987. doi: 10.1016/j.ymgmr.2023.100987 (PMC10320588; doi:10.1016/j.ymgmr.2023.100987)
Supplement: Supplementary file 1 — Supplementary material [file mmc1.docx]

**Supplemental Table 1. Demographics and Baseline Disease Characteristics**

| **Characteristics** | **Patients (n=20)** |
| --- | --- |
| Age at diagnosis, years  n  Mean (SE)  Median (range) | 19 *^a^* 4.01 (1.08) 2.10 (–0.2,*^b^* 16.0) |
| Sex, n (%)  Male  Female | 9 (45) 11 (55) |
| Race, n (%)  White  Asian  Black  Other | 12 (60) 2 (10) 1 (5) 5 (25) |
| History of hydrops fetalis, n (%) | 5 (25) |
| Patients with at least one *GUSB* mutation,*^c^* n (%) | 12 (60) |

*^a^* Age at diagnosis was unavailable for one patient.

*^b^* One patient was diagnosed in utero.

*^c^* For patients without genetic testing, diagnosis was confirmed by GUSB enzyme activity testing. Certain genetic tests were based on data outside of the clinical database (ie, at local sites). Some patients had more than two *GUSB* mutations.

**Supplemental Table 2. Demographics and Baseline Disease Characteristics by Patient**

| **Patient** | **Age at Diagnosis, y** | | **Sex** | **Race** | ***GUSB* Mutation*^a^*** | **Severity of the mutation*^d^*** | **History of Hydrops Fetalis** | **Clinical Manifestations*^b^*** |
| --- | --- | --- | --- | --- | --- | --- | --- | --- |
|  | | *Studies UX003-CL301 and UX003-CL202* | | | | | | |
| 1 | 5.7 | | F | White | ND | NA | No | Skeletal and cardiac manifestations, no/mild cognitive impairment |
| 2 | 5.1 | | F | White | ND | NA | No | Skeletal and cardiac manifestations, cognitive impairment |
| 3 | NA | | F | White | c.1222C>T  (p.P408S)  c.1244C>T  (p.P415L) | Attenuated^38^  *Score 1.0*  Attenuated^38^  *Score 1.0* | No | Skeletal manifestations, no/mild cognitive impairment |
| 4 | 6.1 | | F | Other | c.526C>T  (p.L176F) | Attenuated^39,40^  *Score 1.0* | No | Skeletal and cardiac manifestations, cognitive impairment |
| 5 | 9.8 | | F | White | ND | NA | Yes | Skeletal and cardiac manifestations, severe cognitive impairment |
| 6 | 16 | | M | White | c.1222C>T  (p.P408S)  c.1244C>T  (p.P415L) | Attenuated^38^  *Score 1.0*  Attenuated^38^  *Score 1.0* | No | Skeletal and cardiac manifestations, no/mild cognitive impairment |
| 7 | 0.5 | | F | Other | ND | NA | No | Skeletal and cardiac manifestations, severe cognitive impairment |
| 8 | –0.2 | | F | White | c.1051C>T  (p.H351Y)  c.1289T>C  (p.V429A) | Severe^40^  *Score 1.0*  Unknown  *Score 1.0* | No | Skeletal and cardiac manifestations, no/mild cognitive impairment |
| 9 | 11.5 | | F | White | ND | NA | No | Skeletal and cardiac manifestations, moderate cognitive impairment |
| 10 | 9.9 | | M | Other | ND | NA | Yes | Skeletal and cardiac manifestations, severe cognitive impairment |
| 11 | 0.1 | | M | White | ND | NA | No | Skeletal and cardiac manifestations, mild cognitive impairment |
| 12 | 0.3 | | M | White | c.1051C>T  (p.H351Y)  c.1289T>C  (p.V429A) | Severe^40^  *Score 1.0*  Unknown  *Score 1.0* | No | Skeletal manifestations, no/mild cognitive impairment |
|  | | *Study UX003-CL203* | | | | | | |
| 13 | 0.2 | | M | White | c.88C>T (p.P30S)  c.290G>C (p.G97A) | Unknown^39^  *Score 1.0*  Unknown  *Score 1.0* | No | Skeletal manifestations |
| 14 | 0 | | M | Black | c.148G>T (p.D50Y)  c.1824C>G (p.I608M) | Severe^2^  *Score 1.0*  Unknown  *Score 0.938* | Yes | Skeletal and respiratory manifestations, joint stiffness, developmental delay |
| 15 | 0.3 | | M | Hispanic | c.1A>G  c.1534G>A (p.G512R) | 22 aa missing from signal peptide  Unknown  *Score 1.0* | Yes | Skeletal and respiratory manifestations, joint stiffness, developmental delay |
| 16 | 1.5 | | M | Hispanic | c.526C>T (p.L176F)  c.526C>T (p.L176F) | Attenuated^39,40^  *Score 1.0*  Attenuated^39,40^  *Score 1.0* | No | Skeletal and respiratory manifestations, joint stiffness, developmental delay |
| 17*^c^* | 3.5 | | F | Asian | c.161A>G (p.N54S)  c.1120C>T (p.R374C) c.1136A>G (p.N379S) | Benign  *Score 0.031*  Unknown^40^  *Score 0.994*  Severe^36^  *Score 0.999* | No | Skeletal manifestations |
| 18*^c^* | 1.6 | | F | Asian | c.161A>G (p.N54S)  c.1120C>T (p.R374C)  c.1136A>G (p.N379S) | Benign  *Score 0.031*  Unknown^40^  *Score 0.994*  Severe^36^  *Score 0.999* | Yes | Skeletal manifestations, developmental delay |
| 19 | 2.1 | | F | White | ND | NA | No | Skeletal and respiratory manifestations, joint stiffness, developmental delay |
| 20 | 2.1 | | M | White | c.526C>T (p.L176F)  c.526C>T (p.L176F) | Attenuated^39,40^  *Score 1.0*  Attenuated^39,40^  *Score 1.0* | No | Skeletal manifestations, joint stiffness, developmental delay |

F, female; M, male; NA, not available; ND, not determined.

*^a^* For patients without genetic testing, diagnosis was confirmed by GUSB enzyme activity testing. Certain genetic results were based on data outside the clinical database.

*^b^* Cognitive impairment was per investigator assessment. No formal cognitive testing was performed.

*^c^* Patients 17 and 18 are sisters.

*^d^* Scores of severity (*per PolyPhen-2*) on GUSB missense mutations: Score > 0.908: Probably damaging, Score 0.446 to ≤ 0.908: Possibly damaging, Score ≤ 0.446: Benign.

**Supplemental Table 3. Body Height Z-Scores in Patients with MPS VII Compared with Those of a Healthy Population by Sex and Age Group**

|  | **Male (n=9)** | | |  | **Female (n=11)** | | |
| --- | --- | --- | --- | --- | --- | --- | --- |
| **Age Group** | **n** | **Mean (SD) *Z*-Score** | ***P* Value*^a^*** |  | **n** | **Mean (SD) *Z*-Score** | ***P* Value*^a^*** |
| 0–3 months | 6 | –0.79 (0.83) | 0.067 |  | 4 | 0.13 (1.49) | 0.874 |
| >3–6 months | 5 | –0.82 (1.17) | 0.193 |  | 2 | 1.21 (2.54) | 0.623 |
| >6–9 months | 4 | –1.41 (1.44) | 0.145 |  | 1 | 0.20 | NA |
| >9–12 months | 4 | –1.08 (0.87) | 0.089 |  | 2 | –1.27 (0.75) | 0.252 |
| 1–3 years | 6 | –1.82 (0.85) | 0.003 |  | 5 | –1.41 (0.96) | 0.031 |
| 4–6 years | 3 | –3.11 (1.11) | 0.040 |  | 2 | –2.22 (2.20) | 0.389 |
| 7–9 years | 2 | –4.42 (1.34) | 0.135 |  | 1 | –3.62 | NA |
| ≥10 years | 3 | –3.12 (3.45) | 0.258 |  | 4 | –1.79 (0.92) | 0.030 |

NA, not available; BMI, body mass index.

*^a^* *P* values were derived from t-tests comparing the difference in *Z*-scores between patients with MPS VII and normal patients at each time point.


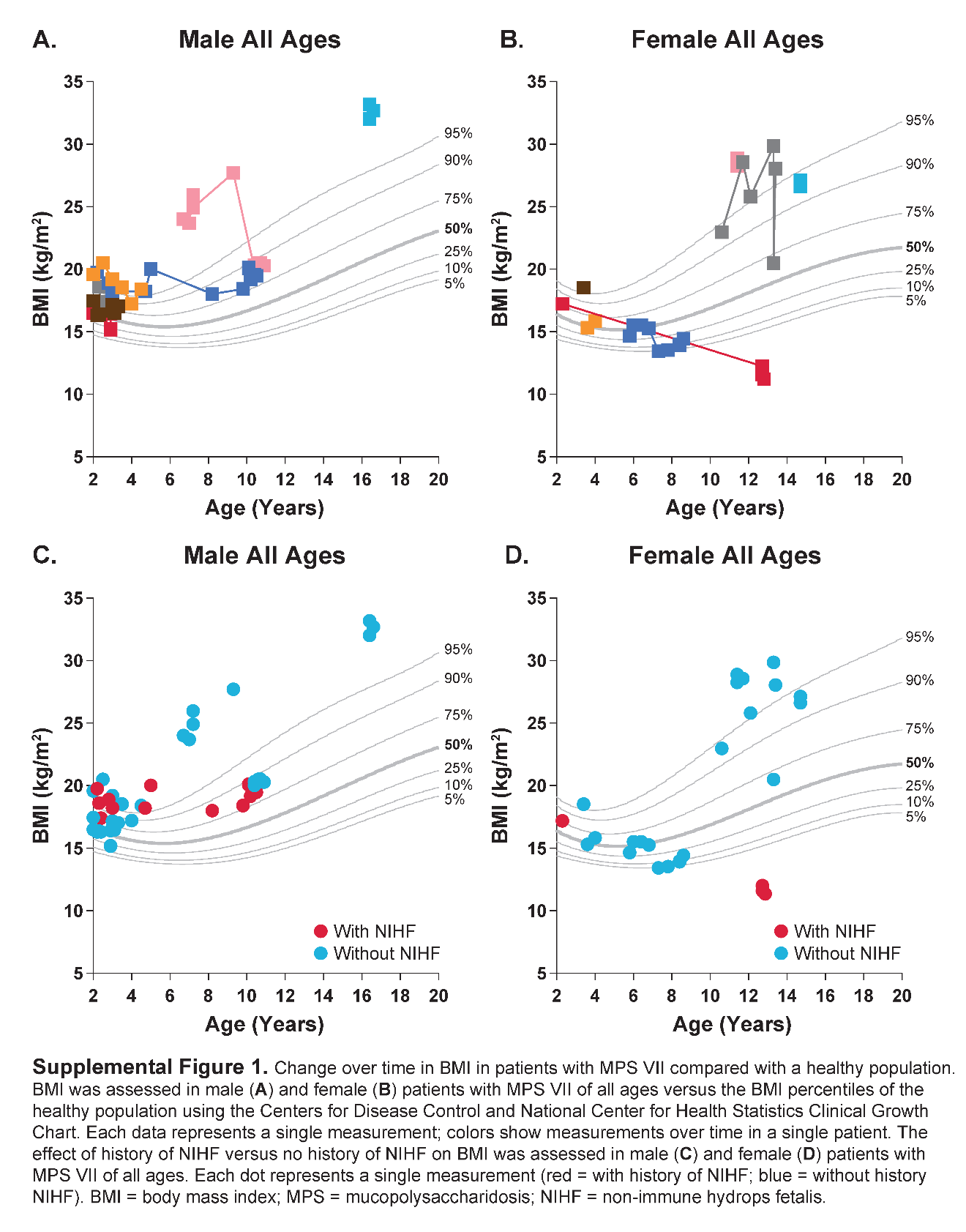


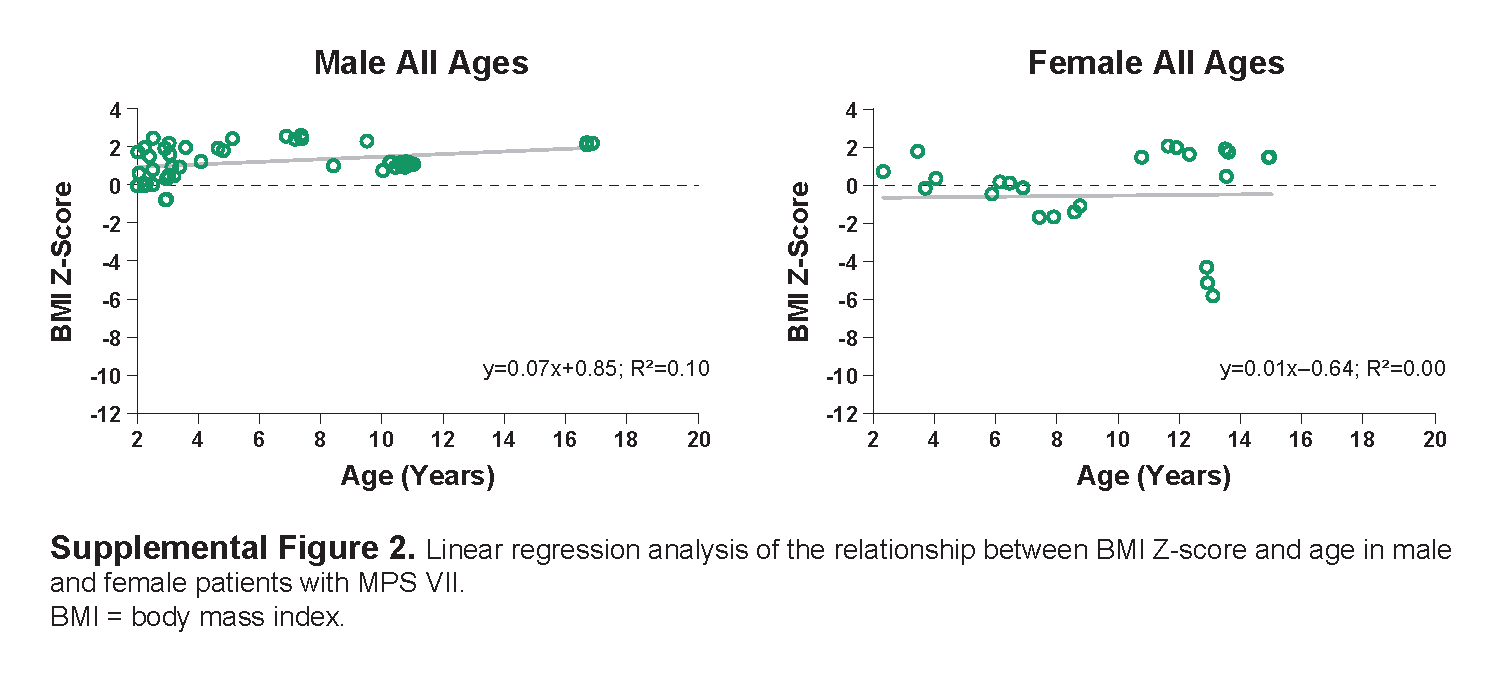


**Supplemental Table 4. Comparison of Body Height, Weight, and BMI by History of NIHF and Age Group in Patients with MPS VII**

|  |  | **History of NIHF (n=5)** |  |  | **No History of NIHF (n=15)** |  |
| --- | --- | --- | --- | --- | --- | --- |
| **Age Group** | **n** | **Mean (SD) *Z*-Score** |  | **n** | **Mean (SD) *Z*-Score** | ***P* Value*^a^*** |
| Body height |  |  |  |  |  |  |
| 0–3 months | 3 | –0.59 (0.97) |  | 7 | –0.36 (1.30) | 0.495 |
| >3–6 months | 2 | –1.76 (1.26) |  | 5 | 0.37 (1.56) | 0.060 |
| >6–9 months | 2 | –1.07 (1.79) |  | 3 | –1.10 (1.59) | 0.461 |
| >9–12 months | 1 | –2.34 |  | 5 | –0.90 (0.54) | NA |
| 1–3 years | 4 | –2.15 (0.96) |  | 7 | –1.34 (0.75) | 0.213 |
| 4–6 years | 1 | –4.19 |  | 4 | –2.39 (1.38) | NA |
| 7–9 years | 1 | –5.37 |  | 2 | –3.54 (0.10) | NA |
| ≥10 years | 2 | –4.23 (3.30) |  | 5 | –1.61 (1.47) | 0.010 |
| Body weight |  |  |  |  |  |  |
| 0–3 months | 4 | –0.11 (1.43) |  | 9 | –0.26 (1.14) | 0.887 |
| >3–6 months | 2 | –4.07 (3.24) |  | 5 | 0.18 (1.12) | <0.0001 |
| >6–9 months | 2 | –0.36 (0.55) |  | 3 | 0.23 (0.34) | 0.588 |
| >9–12 months | 1 | –1.84 |  | 5 | 0.08 (1.40) | 0.193 |
| 1–3 years | 4 | –0.93 (1.27) |  | 10 | –0.16 (1.10) | 0.200 |
| 4–6 years | 1 | –2.82 |  | 5 | –0.94 (1.57) | 0.153 |
| 7–9 years | 1 | –3.44 |  | 3 | –1.52 (2.25) | 0.102 |
| ≥10 years | 2 | –3.85 (0.05) |  | 8 | 0.70 (0.80) | <0.001 |
| BMI |  |  |  |  |  |  |
| 2–3 years | 3 | 1.42 (0.63) |  | 5 | 0.81 (0.94) | 0.629 |
| 4–6 years | 1 | 1.82 |  | 4 | 0.95 (1.28) | 0.927 |
| 7–9 years | 1 | 1.01 |  | 2 | 0.39 (2.93) | 0.799 |
| ≥10 years | 2 | –1.54 (3.86) |  | 5 | 1.69 (0.43) | 0.089 |

F, female; M, male; NA, not available; ND, not determined.

*^a^* For patients without genetic testing, diagnosis was confirmed by GUSB enzyme activity testing. Certain genetic results were based on data outside the clinical database.

*^b^* Cognitive impairment was per investigator assessment. No formal cognitive testing was performed.

*^c^* Patients 17 and 18 are sisters.

**Supplemental Table 5. Publications Reporting Growth Patterns Among Patients with Mucopolysaccharidosis I, II, IVA, VI, and VII**

| **Type of MPS** | **Reference** | **Study Type** | **N** | **Results** |
| --- | --- | --- | --- | --- |
| MPS I | Viskochil D, et al^32^ | Longitudinal registry | 463 | - In patients with severe disease, body height was greater versus reference curves for the first year but decreased by age 4 years and remained decreased |
| MPS I and II | Różdżyńska-Świątkowska A, et al^31^ | Longitudinal | 76 | - Height growth in boys was greater than reference until age 24 months - Height growth slowed at age 2 years for MPS I and age 4 year for MPS II |
| MPS II | Parini R, et al^27^ | Longitudinal | 676 | - Evidence of short stature versus normal appeared by age 8 years - Body weight was higher versus normal until age 9 years and then decreased - BMI was greater than normal until age 14–16 years - Growth velocity was decreased versus normal from 2.5 years onward |
| MPS IVA | Montaño AM, et al^26^ | Longitudinal registry | 354 | - Height growth was normal until age 2 years and decreased beginning at age 4 years - Height velocity decreased after age 1 year - Birth weight was greater than normal but was decreased versus references at age 18 years; however, BMI was greater at age 18 years |
| MPS VI | Quartel A, et al^29^ | Longitudinal | 269 | - After age 4 to 5 years, height growth of rapidly progressing patients decreased versus slowly progression patients - Slow growth of rapidly progressing patients was pronounced after age 10-12 years |
| MPS VII | Montaño AM, et al^2^ | Longitudinal | 12 | - Normal height growth until aged 18-24 months, followed by rapid decline - Short stature was more pronounced in girls than in boys - BMI was typically normal |
